# Community first responders and responder schemes in the United Kingdom: systematic scoping review

**DOI:** 10.1186/s13049-017-0403-z

**Published:** 2017-06-19

**Authors:** Viet-Hai Phung, Ian Trueman, Fiona Togher, Roderick Orner, A. Niroshan Siriwardena

**Affiliations:** Community and Health Research Unit, School of Health and Social Care, University of Lincoln, Brayford Campus, Lincoln, LN6 7TS UK

**Keywords:** First responders, Prehospital care, Urgent care, Basic ambulance care

## Abstract

**Background:**

Community First Responder (CFR) schemes support lay people to respond to medical emergencies, working closely with ambulance services. They operate widely in the UK. There has been no previous review of UK literature on these schemes. This is the first systematic scoping review of UK literature on CFR schemes, which identifies the reasons for becoming a CFR, requirements for training and feedback and confusion between the CFR role and that of ambulance service staff. This study also reveals gaps in the evidence base for CFR schemes.

**Methods:**

We conducted a systematic scoping review of the published literature, in the English language from 2000 onwards using specific search terms in six databases. Narrative synthesis was used to analyse article content.

**Results:**

Nine articles remained from the initial search of 15,969 articles after removing duplicates, title and abstract and then full text review.

People were motivated to become CFRs through an altruistic desire to help others. They generally felt rewarded by their work but recognised that the help they provided was limited by their training compared with ambulance staff. There were concerns about the possible emotional impact on CFRs responding to incidents. CFRs felt that better feedback would enhance their learning. Ongoing training and support were viewed as essential to enable CFRs to progress. They perceived that public recognition of the CFR role was low, patients sometimes confusing them with ambulance staff. Relationships with the ambulance service were sometimes ambivalent due to confusion over roles. There was support for local autonomy of CFR schemes but with greater sharing of best practice.

**Discussion:**

Most studies dated from 2005 and were descriptive rather than analytical. In the UK and Australia CFRs are usually lay volunteers equipped with basic skills for responding to medical emergencies, whereas in the US they include other emergency staff as well as lay people.

**Conclusion:**

Opportunities for future research include exploring experiences and perceptions of patients who have been treated by CFRs and other stakeholders, while also evaluating the effectiveness and costs of CFR schemes.

## Background

A Community First Responder (CFR) “*is a member of the public who receives basic emergency care training and volunteers to help their community by responding to appropriate medical emergencies while an ambulance is en route*” [1]. They complement the work of the ambulance service. Their work is particularly valuable in rural communities, where it might take ambulances longer to reach medical emergency situations.

Community First Responder schemes have been providing prehospital emergency care since the 1990s, enabling patients to receive early medical attention while awaiting an ambulance response [2]. The ambulance service deploys an estimated 2,500 CFR schemes, with over 11,000 volunteers in the United Kingdom [1, 3]. They are usually charities, either independent or run through ambulance trusts [4]. Currently, no national standards exist concerning CFR service provision, training and support of volunteers or quality of services provided. Local schemes have developed independently of each other and reflect each area’s priorities. Many CFR schemes only respond to cardiac events, whilst others may also attend road traffic collisions and trauma incidents. Such diversity of provision also carries varying degrees of training and support of volunteers which could impact on effectiveness, safety and retention of personnel [1].

Some UK regions, such as the East Midlands, have both independent CFR schemes and schemes run by ambulance services. For example, Lincolnshire Integrated Voluntary Emergency Service (LIVES) is an independent voluntary scheme working collaboratively with but not managed by the regional ambulance service, whereas the CFR scheme in Nottinghamshire is run by the ambulance service. Both, like many other CFR schemes, have volunteers trained up to ‘first person on scene’ level [3].

The Government has called for greater co-ordination and collaboration between ambulance services, the 111 call service, which provides advice for urgent but non-emergency cases, urgent care and out-of-hours services in *The NHS 5 year forward view* [5]. Such changes are likely to affect CFR schemes within ambulance trusts and CFR schemes working with other agencies to ensure a more integrated and needs-led service [6, 7]. Therefore, it is timely to evaluate the CFR role and service provision and explore their potential for future development.

Research on the benefits of CFR schemes to both patients and ambulance services for health outcomes and ambulance response times have been published for other countries [8] but there has been no review of published literature on CFR schemes in the UK. This is the first systematic scoping review of UK literature on CFR schemes, which identifies the reasons for becoming a CFR, requirements for training and feedback and confusion between the CFR role and that of ambulance service staff. This study also reveals gaps in the evidence base for CFR schemes.

## Methods

We aimed to map existing published literature relating to current UK-based CFR schemes in order to identify gaps for future research to explore. To do so, we conducted a systematic scoping review of published research on CFR schemes and CFRs including any interventions, comparisons and outcomes. The purpose of the study was to understand, map and synthesise the range of published literature, regardless of quality [9].

### Inclusion criteria

The inclusion criteria for selecting publications were that they had to be published in English and from the year 2000 onwards in order to reflect current UK CFR schemes. All studies had to be UK-based, so non-UK studies were excluded. The final agreed search terms were as follows:

“emergency responder*” OR “lay responder*” OR “first person on scene” OR “community first respon*” OR “community respon*” OR “first respon*” OR “first-respon*” OR “Community” AND “first” AND “responder”

### Data sources

The following databases were searched: CINAHL; MEDLINE; PsycINFO; Applied Social Sciences Index and Abstracts (ASSIA); International Bibliography of the Social Sciences (IBSS); Published International Literature on Traumatic Stress (PILOTS).

### Search strategy

**Fig. 1:**
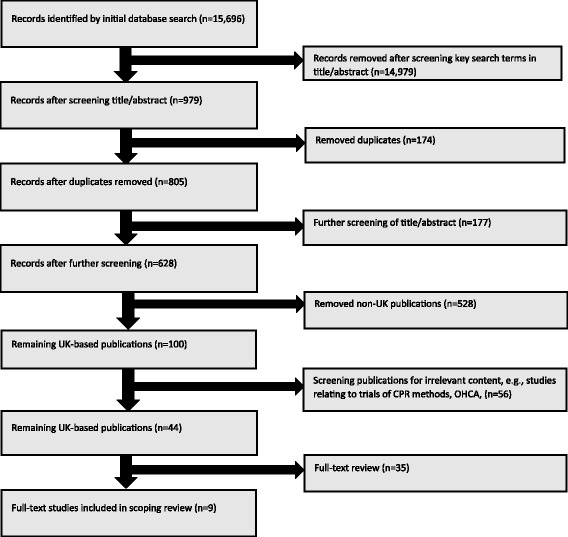
Study flowchart

Search results were scanned individually for relevance. Selection at this stage included direct relevance to the research question (i.e. included key search terms in title/abstract) or potential usefulness as background information. Articles deemed relevant from each database were exported into an individual EndNote library. This resulted in 979 articles, of which 174 duplicates were removed, leaving 805 articles for screening. Screening by title and abstract excluded a further 177 articles. Since we wished to focus on UK-based CFR schemes, of the remaining 628 articles, 528 were rejected because they referred to schemes outside the UK. The 100 papers left included 56 studies of CPR methods, mass casualty terror acts, etc., which were removed. Two researchers (IT and FT) conducted a full-text review of the remaining 44 articles, in which a further 35 publications were excluded. This left nine publications in the scoping review (Fig. 1). Data were extracted for each study describing ‘aims and objectives’, ‘sample population’, ‘methods and ‘results’. Scoping reviews by their nature do not exclude studies with higher risk of bias, so no risk of bias analysis was undertaken.

## Results

Of these nine publications, one was a systematic review, four were qualitative studies, three used quantitative methods, and another employed a mixed-methods approach (Table 1).

**Table 1:** Summary of included studies

Study	Aims and objectives	Sample population	Methods	Results
Davies et al. (2008) [10]	To investigate the psychological profile of first responders to gain insight into possible factors that might protect them against such reactions.	First responders in a community scheme in Barry, South Wales.	In depth semi-structured interviews with six subjects were analysed using Interpretive Phenomenological Analysis (IPA).	CFRs were motivated by a sense of duty to their community. They found it rewarding when they contributed positively to a patient’s outcome. They felt it was important to understand their role and the limitations on it. CFRs described an emotionally detached state of mind, which helped them remain calm in these potentially stressful situations
Dennis et al. (2013) [14]	To investigate which categories of Emotional Support messages could be used to support a CFR when they are experiencing different kinds of stress in the field.	20 participants from Amazon’s Mechanical Turk service. For this validation experiment (HIT), participants had to be based in the US and have an acceptance rate of 90% (90% of the work they do is accepted by other requesters had to be rated as good quality) and were paid $0.50 (US).	As before, the validation experiment took the form of an online questionnaire administered on Mechanical Turk, with the same participation criteria. Participants were paid $0.50 and we received 40 responses. Participants were asked to indicate their gender, their age from a range and indicate if they were a health professional. 55% were female (45% male), 22.5% were aged 15-25, 45% 26-40, 27.5% 41-65 and 5% over 65. 5% of participants were healthcare professionals.	*Directed Action* was the most popular category for Mental Demand (where the CFR needs to think), Temporal Demand (time pressure), Frustration, Distraction and Isolation. *Reassurance* was the most popular category for the remaining stressors – Physical Demand and Emotional Demand. *Praise* was also popular for Mental Demand, Physical Demand, Frustration, Distraction and Isolation. *Emotional Advice* appears to be much less popular, only used by few participants in Frustration, Distraction, Emotional Demand and Isolation. *Emotional Reflection* was only used for Frustration and Distraction.
Faddy and Garlick (2005) [16]	This review aimed to determine whether 50% nitrous oxide was safe for use by first responders who are not trained as emergency medical technicians.	From the electronic search of the Medline and EMBASE databases we identified 1,585 citations that matched the search criteria. These were screened for potentially relevant studies. A total of 158 abstracts were retrieved for more detailed evaluation, of which 33 described studies that were potentially relevant to this systematic review. These studies underwent critical appraisal. Twelve studies satisfied all subject and methodology criteria and were subsequently included in the review	One reviewer performed all of the literature searches. The reviewer searched the Medline (1966–Oct 2001) and EMBASE (1985—Oct 2001) databases, using an optimally sensitive search strategy, for relevant studies comparing 50% nitrous oxide with placebo or other analgesic agents in the prehospital setting. Again, no studies in the prehospital setting were found. Consequently, a broader search was performed to find randomised controlled trials from a wide range of clinical settings. Reference lists cited in original articles were examined for relevant studies not identified by the literature search.	Two studies assessed drowsiness in 135 patients treated with 50% nitrous oxide or placebo [16, 18]. The pooled result of these studies showed that 3% of patients treated with nitrous oxide analgesia and 4% of patients treated with placebo experienced drowsiness, indicating that drowsiness was probably unrelated to nitrous oxide inhalation (RD 21%, 95% CI 27% to 5%, *p*=0.8). The incidence of hypotension in patients who were treated with nitrous oxide was lower than in patients treated with intravenous midazolam and pethidine (14%).
Farmer et al. (2015) [12]	This article explored what happened, over the longer term, after a community participation exercise to design future rural service delivery models, and considered perceptions of why more follow-up actions did or did not happen.	22 citizens in three Scottish communities (healthcare practitioners, managers and policymakers) all of whom were involved in, or knew about, the original project.	In-depth interviews to capture stakeholders' views. A semi-structured topic schedule was developed to ensure a similar approach across sites.	All citizen participants of community C described the first responder scheme and its establishment during or just after Remote Service Futures, facilitated by training from the Scottish Ambulance Service. They said new volunteers were now needed. Two participants reported that, following the initial scheme establishment and training, there had been little follow-up by staff of any health-related service and that current first responders had not received ongoing training, leaving them feeling unsupported. Community members felt let down by state authorities and questioned whether volunteering should continue as it might be hindering provision of a statutory service.
Harrison-Paul et al. (2006) [15]	To explore the experiences of lay people who have been trained to use automatic external defibrillators. The research questions were: (1) How can training courses help prepare people for dealing with real life situations? (2) Who is ultimately responsible for providing critical incident debriefing and how should this be organised? (3) What is the best process for providing feedback to those who have used an AED?	53 participants, some of whom had been given training to use defibrillators and others who delivered the training. Locations included airports, railway stations, private companies and first responder schemes. Geographically, the study covered Nottinghamshire, Lincolnshire, Yorkshire, Staffordshire, Essex and the West Midlands in the UK.	Semi-structured, qualitative interviews.	Most people believed scenarios based within their place of work were most useful in preparing for ‘real life’. Many people had not received critical incident debriefing after using an AED. There were a variety of systems in place to provide support after an incident, many of which were informal.
Kindness, et al. (2014) [1]	To further understand the demands and stressors experienced by CFRs.	An online survey using a modified NASA-TLX scoring system was sent to 535 Community First Responders in Scotland.	CFRs were asked to gauge the demands and stressors experienced during a 'typical' and their 'most stressful' callout, what would be the biggest cause of stress if present and the most stressful time -period during callouts.	88 CFRs started the survey with 40 continuing to completion. Frustration that the CFR could not help the patient more was considered to be the biggest stressor for both a typical and a most stressful callout. Emotional demand was the most present demand in a typical callout and mental demand in the most stressful callout. If present, loneliness and isolation was deemed to be the biggest cause of stress for CFRs. Prior to arrival at scene was the most stressful time.
Roberts, et al. (2014) [4]	To capture the CFR activity data at the same time as gathering in depth, robust qualitative material. Included were stakeholder interviews (e.g. with representatives of national and local government, health authority, health professionals, and community members), and focus groups with individual CFRs.	Participants included purposively selected representatives from the Scottish Government (in the area of performance management for emergency medicine), Scottish Ambulance Service personnel, community engagement representatives from the Scottish Health Council, local after-hours service managers and General Practitioners (GPs).	Study 1 (March 2009 – December 2010) evaluated the introduction of a CFR scheme in an isolated region with difficulties created by geography where the drive time to the nearest hospital with a major A & E department was more than 90 minutes. Study 2 (October 2010 – September 2011) investigated the contribution of six CFR schemes in urban, suburban and remote Scottish settings. Data collection during both studies were mixed methods. Routine anonymised data provided by Scottish Ambulance Service about callouts were analysed. These were supplemented by face-to-face or telephone interviews, as well as CFR focus groups.	CFRs were enthusiastic about contributing to their community. Supportive relationships amongst volunteers within their schemes and support from the wider ambulance service staff were reported. SAS employees and CFRs agreed on the scope of practice of CFRs’ emergency response duties, but community members were confused about the CFRs role. During the focus groups, CFRs were concerned that community members lacked knowledge about the response process, particularly CFRs only responding once an ambulance has been dispatched. CFRs perceived confusion in communities about reasons for introducing schemes. All CFR volunteers in all schemes thought that more publicly available information describing the CFR role and “the point that the ambulance is on its way” would help community members understand why CFRs volunteer and this may impact upon acceptance. A commonly raised theme among CFRs and ambulance personnel was that while volunteers must act professionally according to a formal code of conduct and protecting patient information, they do not have the same emergency professional qualification that their colleagues have. CFRs felt that the lack of feedback about how patients fared was difficult to deal with. They were not formally informed about what happened to people after their first response assistance. This was challenging because they worked in the locality and may know the patient, their family or friends. Confidentiality prevented them from asking and yet they were often interested and concerned about fellow community members.
Seligman, et al. (2015) [13]	The paper discusses the experience of launching the student first responder (SFR) scheme across three counties in the Thames Valley.	Students participating in the SFR scheme in the Thames Valley region. The size of the SFR group as of August 2014 was 72.	Data on the number of students participating in the SFR scheme were obtained from SCAS records. SCAS data were also obtained to determine the number and type of incidents to which SFRs were being dispatched. An electronic survey was carried out in April–May 2015 of all Foundation Doctors who had been members of this SFR scheme during their time at medical school.	In the first 15 months of operation (June 2013– August 2014), SFRs were dispatched to 343 incidents. The Most common types of calls that they attended to were: other; respiratory emergencies; non-traumatic falls; and gastrointestinal emergencies.
Timmons and Vernon-Evans (2012) [11]	To understand why people volunteer for, and continue to be active in CFR groups.	CFR volunteers from one English region. Although, as a qualitative study, a statistically representative sample was not needed, the geographical region was intended to generate a mixture of CFR groups from urban, suburban and rural communities and being mixed in terms of socio-economic status. The different locations help to create a balanced sample.	Given that the participants are volunteers who only meet infrequently as a group, focus groups were the most efficient and cost-effective way of collecting data.	The most common route was finding out about CFRs through an advertisement in the local newspaper. Many participants joined to ‘get involved’ or ‘get out in the community’, as each first responder group is a local charity and relies on volunteers and financial support from within the community. A key factor in getting people to volunteer, but more importantly to stay, was the flexibility of the role and the nature of the role itself. Participants valued their role as an assistant to the paramedic. The experiences with the ambulance service had not always been good. The flexible nature of the CFRs’ commitment may have played a part in this. CFR groups rely on money from the local community and they spend a lot of time raising funds at local events. Participants highlighted the significance of the community supporting their local group, and how locals like to see good work being done that directly affects them and their community.

We used a narrative approach to summarise the main findings in themes described below.

### Motivations and reasons to become a CFR

Several studies showed that volunteers cited altruistic reasons for becoming CFRs [10, 11]. Becoming a CFR was often seen as a way of giving something back to the community by helping others [4, 10–12]. The role was also seen as a way of enhancing employability within the ambulance care sector [13]. Some CFRs joined because they were already healthcare professionals who felt that it provided a good learning experience for them in a different setting [13].

### Experiences of being a CFR

CFRs felt their role was rewarding, although they expressed a need for praise for the work they did [4] and a concern about the limited opportunities for operational debriefing on their activities [10, 14, 15] CFRs felt they were limited in what they could do because they lacked the skills of paramedic staff. [1, 12] In some instances, this manifested in a concern that they were not doing the right thing [1], while some felt they could and should be able to do more to help patients [16].

### Training

We found no evidence around the content of the initial training of CFRs, but this identified the need for research on the requirements for ongoing training and support. Previous studies pointed to a mandatory period of experience required of CFRs before they were allowed to progress to higher levels of expertise [16]. CFRs felt that ongoing training was essential to enable them to progress.[12, 15]. However, retraining and keeping up to date in a timely manner was considered difficult [1, 15]. CFRs expressed concerns that despite the ongoing training, this training would become less relevant if they had not been called out to patients [1, 12, 15] Furthermore, CFRs felt that provision of training demonstrated how their organisation valued the contribution they made to patient outcomes [12]. Conversely, a lack of training led to frustration among CFRs about not having the skills required to help patients [1].

In terms of the types of training that CFRs undertook, scenario-based training was considered to be the most effective [15]. Training was sometimes considered to be too focused on skills, with a greater need to emphasise the emotional side of being a CFR [1, 15].

### Patient outcomes and feedback

CFRs were not usually given feedback about patients they had attended. This was something that CFRs wished to see change [1, 15]. They felt that evidence of improved patient outcomes could enhance their profile in the local community and offer greater personal recognition of the work they did [4, 12]. Even without formal feedback mechanisms, some CFRs derived satisfaction from contributing positively to patient outcomes [10].

### Public understanding of CFRs

There was a low level of public recognition of the CFRs’ role. There was perceived public confusion about how their role related to that of the ambulance service. [4] For example, the public were concerned that CFRs may adopt roles traditionally associated with ambulance staff, reducing the effectiveness of the ambulance service [12]. Recruitment was often poor in areas where the ambulance service was perceived to be performing well [12]. In order to tackle low levels of recognition, CFR programmes felt they needed to work closely with stakeholders and consumers to improve the way they publicised themselves [4, 11].

### Relationship between CFRs and the ambulance service

There was a perception of ambivalence in the relationship between CFRs and the ambulance service [12]. This stems partly from some confusion over accountability between the ambulance service and CFR schemes. Some CFRs felt undervalued by ambulance service staff [1].

### Emotional impact

Much of the literature around CFRs centred on the emotional impact of the role. Despite call handlers giving CFRs an indication of the nature of the incidents that they were responding to, CFRs maintained a flexible approach on reaching the patient [10], because what they found at the scene might have been very different to what had been communicated by call handlers. The role also necessitated an ability to switch off from the often traumatic nature of the incidents they attended to [1, 10] There were particular concerns about the potential for lone working to have a high emotional impact [14]. That said, some CFRs valued having support mechanisms to call upon when needed [1, 14].

### Suggestions for improvement

CFRs expressed a need for ongoing training and support in order for them to feel valued and appreciated. To do this, it was felt that shared governance, collaboration with statutory providers to fully fund training, and assistance with resources would greatly help [11].

In terms of how CFR schemes develop further, there was strong support for local autonomy together with greater collaboration between schemes [11, 15]. A key strength of CFR schemes was that they reflected local needs and demands. If they are to be rolled out more widely, then new schemes could follow best practice from existing schemes that have been shown to work effectively. This potentially conflicted with the suggestion for nationwide minimum standards for CFRs [2].

## Discussion

### Main findings

People became CFRs mainly to help others and put something back into their communities. CFRs also wanted to be appreciated and recognised for their work, perhaps through integrating formal feedback mechanisms into practice. Both are relevant considerations for CFR schemes needing to recruit and retain volunteers.

CFRs particularly valued scenario-based training which they felt would most effectively improve their range of skills. Maintaining the realism of scenario-based training, as well as encouraging CFRs to improve their skills will enable them to attend to a greater range of incidents, which is what they want.

CFRs valued the flexibility and availability of support mechanisms to help them cope with the stressful incidents, which they inevitably have to attend to from time to time. Nevertheless, the scoping review raised awareness of some of the known risks associated with attending to particular incidents. It also identifies the stress factors of other, non CFR-related, pressures a responder may struggle with.

While this is a UK-based scoping review, it is important to draw some comparisons with how CFR schemes work in other countries. In the UK, CFRs are volunteers equipped with some basic skills in life support to enable them to respond to medical emergencies. Their purpose is to do the preparatory work at the scene prior to ambulance service staff arriving. In the US, first responders can include Police Officers, firefighters and other emergency services staff, as well as lay people [17, 18]. Australian volunteer response resembles the UK model in that it relies on lay people volunteering to help emergency services respond to incidents [19].

### Strengths and limitations

The precise search criteria applied to this scoping review produced nine UK-based publications. The low number may be because research into CFRs is relatively recent, with most studies being from 2005 onwards. Because much of the research into CFRs was recent, the included publications tended to be more descriptive than analytical. Indeed, the existing literature mainly comes from the perspective of ‘experts’.

### Implications for policy and research

Future research should explore the perspectives of the patients who had received care from CFRs as well as that of CFRs, commissioners, policymakers and academics. Perceptions of patients are important because there is limited understanding of patients’ experiences of the service as well as limited public awareness and understanding of what CFRs do.

Patients were sometimes unable to distinguish between CFRs and ambulance crews. In some instances, patients were less concerned about the respective roles of each but instead were grateful and reassured about the presence of someone with expertise and skills and to help them in a highly stressful situation. Some CFR schemes had attempted to rectify this situation by raising awareness in their communities about how they operated.

Clarifying the role of the CFR is important as their relationship with the ambulance service was sometimes mixed. Sometimes, ambulance crew were grateful for the preparatory work that CFRs did prior to their arrival. In other instances, staff from ambulance and other statutory services viewed CFRs with suspicion because of a lack of understanding about when the CFR’s role ends and the ambulance crew’s begins. This suggests that future research could usefully explore the perceptions of ambulance service staff towards CFRs.

This tension and confusion around roles is partly reflected in the low public awareness about differences between CFRs and ambulance crews. To address this confusion, there needs to be greater clarity over the roles of ambulance staff and CFRs.

There is an opportunity to explore the proportion of ambulance service cases that are attended to by CFRs and the contribution that CFRs make to response time targets or patient outcomes. Outcomes research could focus on overall caseload or specific time-sensitive conditions, such as cardiac arrest. The scoping review identifies that these are matters of policy which should be clarified in operational practice. Once these are in place, research might generate an evidence base upon which decisions can be made about the formal and informal status of CFR services and their role within the communities they serve.

The local nature of CFR schemes means that by definition, they are driven by local contextual factors, such as demographics, geography, demand and available skills sets. It might be more appropriate to have minimum standards of training for CFRs. Urban and rural service settings may require different operational policies, training priorities, safety measures and follow-up arrangements for CFRs. Outcome standards could vary between local schemes to reflect such local factors. Local CFR schemes need to be clear about what the priorities are in their area. This should then inform their desired outcomes and objectives. Once local schemes are clear about their desired outcomes and objectives, then they can have a better idea of what role their volunteers should have and tailor their training programmes accordingly. Future research can clarify the extent to which aims and objectives are locally defined as well as how CFR schemes operate to give a more nuanced perspective about the links between local provision and local needs. Once more is known about how schemes operate, there is greater potential for best practice to be shared, especially between localities with similar demographics, context and need.

CFRs felt strongly about the effectiveness of scenario-based training and the desirability of having formal feedback mechanisms, therefore, it would be helpful to involve them in deciding how these might be incorporated into local schemes.

## Conclusions

This scoping review has identified and highlighted numerous opportunities for future research. These include: exploring patients’ experiences and other stakeholder views; evaluating the effectiveness; costs; and support needed to ensure quality of CFR schemes. Such evidence may inform the way that CFR schemes develop services in future as well as training mechanisms to ensure that CFRs feel valued and well-supported. Further understanding of the stressors associated with the role should assist in limiting turnover rates. This will help to secure the long-term future of the CFR schemes and the vital services they provide in complementing the statutory emergency care services.

## Abbreviations

ASSIA: Applied social sciences index and abstracts
CFR: Community first responder
IBSS: International bibliography of the social sciences
LIVES: Lincolnshire integrated voluntary emergency service
PILOTS: Published International literature on traumatic stress
UK: United Kingdom
